# Targeted CRISPR activation is functional in engineered human pluripotent stem cells but undergoes silencing after differentiation into cardiomyocytes and endothelium

**DOI:** 10.1007/s00018-023-05101-2

**Published:** 2024-02-19

**Authors:** Elaheh Karbassi, Ruby Padgett, Alessandro Bertero, Hans Reinecke, Jordan M. Klaiman, Xiulan Yang, Stephen D. Hauschka, Charles E. Murry

**Affiliations:** 1 Institute for Stem Cell and Regenerative Medicine, University of Washington, Seattle, WA 98109 USA; 2 Center for Cardiovascular Biology, University of Washington, Seattle, WA 98109 USA; 3 Department of Laboratory Medicine and Pathology, University of Washington, Seattle, WA 98195 USA; 4 Division of Cardiology, Department of Medicine, University of Washington, Seattle, WA 98195 USA; 5 Department of Bioengineering, University of Washington, Seattle, WA 98195 USA; 6 Center for Translational Muscle Research, University of Washington, Seattle, WA 98109 USA; 7 Department of Biochemistry, University of Washington, Seattle, WA 98109 USA; 8 Present Address: Molecular Biotechnology Center “Guido Tarone”, Department of Molecular Biotechnology and Health Sciences, University of Torino, Torino, 10126 Italy

**Keywords:** Stem cell engineering, CRISPR activation, Genome engineering, Transgene silencing

## Abstract

Human induced pluripotent stem cells (hiPSCs) offer opportunities to study human biology where primary cell types are limited. CRISPR technology allows forward genetic screens using engineered Cas9-expressing cells. Here, we sought to generate a CRISPR activation (CRISPRa) hiPSC line to activate endogenous genes during pluripotency and differentiation. We first targeted catalytically inactive Cas9 fused to VP64, p65 and Rta activators (dCas9-VPR) regulated by the constitutive CAG promoter to the *AAVS1* safe harbor site. These CRISPRa hiPSC lines effectively activate target genes in pluripotency, however the dCas9-VPR transgene expression is silenced after differentiation into cardiomyocytes and endothelial cells. To understand this silencing, we systematically tested different safe harbor sites and different promoters. Targeting to safe harbor sites *hROSA26* and *CLYBL* loci also yielded hiPSCs that expressed dCas9-VPR in pluripotency but silenced during differentiation. Muscle-specific regulatory cassettes, derived from cardiac troponin T or muscle creatine kinase promoters, were also silent after differentiation when dCas9-VPR was introduced. In contrast, in cell lines where the dCas9-VPR sequence was replaced with cDNAs encoding fluorescent proteins, expression persisted during differentiation in all loci and with all promoters. Promoter DNA was hypermethylated in CRISPRa-engineered lines, and demethylation with 5-azacytidine enhanced dCas9-VPR gene expression. In summary, the dCas9-VPR cDNA is readily expressed from multiple loci during pluripotency but induces silencing in a locus- and promoter-independent manner during differentiation to mesoderm derivatives. Researchers intending to use this CRISPRa strategy during stem cell differentiation should pilot their system to ensure it remains active in their population of interest.

**Supplementary Information:**

The online version contains supplementary material available at 10.1007/s00018-023-05101-2.

## Introduction

CRISPR technology has developed into a versatile tool to systematically test the function of genes and transcripts in vitro and in vivo for gain- and loss-of-function studies. Applications include understanding gene functions, regulating genomic loci, programming cell states, and performing high throughput genome-wide forward screens [1, 5]. Protocols to implement such applications are becoming widely available. CRISPR activation (CRISPRa) has emerged as a flexible approach to upregulate genomic loci. The most common variations of the CRISPRa system [14] comprise a catalytically inactive Cas9 (dCas9) fused directly to transcriptional activators (TA) or recruiting them through RNA scaffolds. By the addition of guide RNAs (gRNAs), these dCas9-TA proteins can be directed to target loci to promote the recruitment of transcriptional machinery. In the regulation of gene expression, dCas9-TA can be recruited to promoters to effectively upregulate expression of protein-coding and non-coding regulatory RNAs from specific loci. This induces expression of mature transcript isoforms, with post-transcriptional processing comparable to that which arises with natural promoter activation.

Traditional gain-of-function experiments entail transfection or transduction of exogenous DNA, which allows for robust expression. These approaches have their limitations, however. There can be large heterogeneity in the cell population; not all cells are transduced, while others receive “supraphysiological” copies of the introduced DNA. Cell cycle state may influence transduction efficiency, with dilution of episomal DNA in dividing cells [21] or poor expansion of cells with viral integration [18, 20]. Furthermore, in the case of cardiomyocytes, transfection is not efficient [57], and viral strategies are typically required to introduce genes [46, 52]. In all cases, one needs to know the specific sequence desired for expression, which can limit complexity in alternatively spliced transcripts. In contrast to bulk transfection, a stably engineered cell line can provide a homogenous clonal cell population, giving consistency between biological experimental replicates. Stable lines typically afford long-term expression and minimize off-target effects due to random integration sites and variations in copy number.

Access to human primary cells is limited, whereas human induced pluripotent stem cells (hiPSCs) provide a limitless supply of cells that can be used to study human biology, making mechanistic basic biology studies feasible. Moreover, these in vitro models can be genetically engineered to model human genetics or express reporters that will aid in studying their biology. CRISPRa can be applied to study biology of pluripotency [56], differentiation pathways [11] and specialized cell derivatives [60]. Such gain-of-function studies require a homogenous cell population that can be expanded, differentiated, and express functional CRISPRa effectors in undifferentiated and differentiated states [41]. An important consideration is the persistent activity of the transgenes across stages of development as cells transition from one cell type to another. More importantly, a targeted approach is advantageous, as random integration may interfere with differentiation into various lineages, and can be subject to silencing and not reproducible [50]. Several groups have generated human CRISPRa stem cell lines, yet the functionality of these lines has not been characterized across differentiation in depth in a variety of human stem cell-derived lineages [22, 24, 53, 60].

Here, we generate a constitutive CRISPRa induced pluripotent stem cell line, targeting dCas9 fused to VP64, p65 and Rta (dCas9-VPR) [17] in WTC11 hiPSCs, that can be readily differentiated into cardiomyocytes (hiPSC-CM). Our goal is to generate a CRISPRa cell model that can be applied to activate large structural genes so as to better understand cardiac function or regulatory genes to dissect cardiac development and differentiation pathways, to improve cardiac regenerative strategies, and to serve as a screening platform. We first targeted the *AAVS1* safe harbor locus. However, we observed that with differentiation of CRISPRa stem cells, the dCas9-VPR transgene is silenced, precluding activation of target genes in hiPSC-CMs. We turned to alternative approaches, including targeting two additional safe harbor loci (human *ROSA26* and *CLYBL*) as well as implementing muscle-specific promoters. These, too, resulted in silencing of the transgene after differentiation. This locus- and promoter-independent silencing demonstrates challenges with using CRISPRa in hiPSC-derived cardiomyocytes and endothelial cells, and perhaps, in other cell types differentiated from pluripotent stem cells.

## Results

### Targeting the AAVS1 safe harbor locus for constitutive CRISPRa expression

**Fig. 1:**
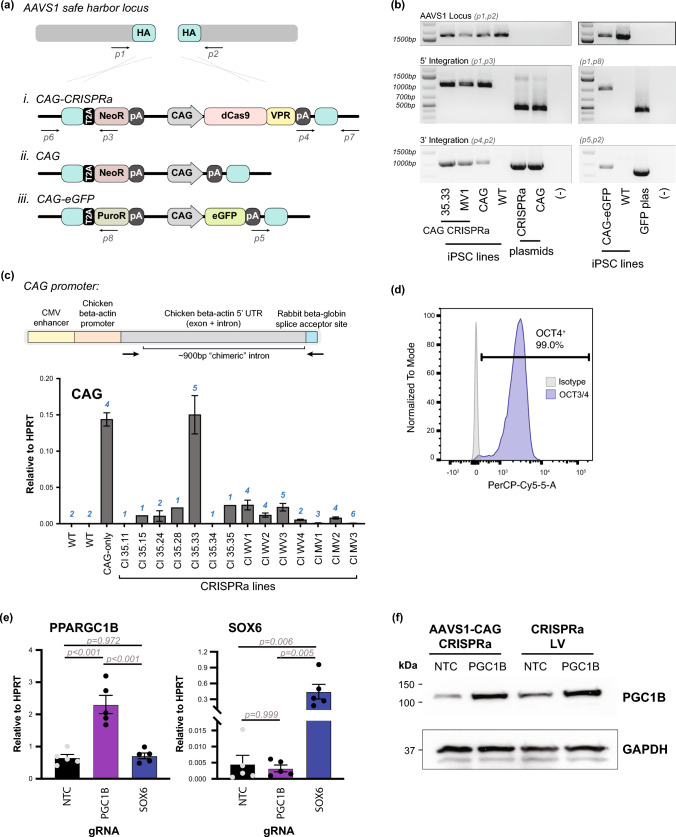
Generation of constitutive CRISPRa WTC11 stem cell line. **a** Schematic of targeting strategy to the *AAVS1* safe harbor locus between exons 1 and 2 (chr19:55115764–55115767). The following transgenic lines were generated: (*i*) dCas9-VPR driven by the CAG promoter (*ii*) CAG promoter only (*iii*) eGFP driven by CAG; all lines also contained antibiotic resistance as a selection marker (either NeoR or PuroR), with expression driven by the endogenous locus. *HA: homology arm; NeoR: neomycin resistance; PuroR: puromycin resistance; T2A/P2A: self-cleaving peptide; pA: poly A.* **b** Genotyping of WTC11 *AAVS1*-CAG cell lines. Primers used are noted and target sites are shown in (**a**). Genomic DNA from wild type (WT) hiPSCs and donor plasmids used for cell line generation were used as control template DNA. **c** Primers flanking the CAG promoter chimeric intron are used to measure mature transcripts and expression from this locus. Quantitative PCR is used to measure CAG transcripts, indicative of transgene expression across different CRISPRa clones. Expression is normalized to HPRT housekeeping gene. *n = 1–6 biological replicates (indicated for each sample).* **d** OCT4 flow cytometry demonstrates pluripotency state. Gating performed using isotype control antibody. **e** *AAVS1*-CAG-CRISPRa stem cells (Clone 35.33) can induce mRNA expression of gRNA-targeted genes. Expression is normalized to HPRT. One-way ANOVA, followed by post-hoc Tukey-Kramer test, was performed to calculate statistical significance. *n = 5 biological replicates.* All error bars represent SEM. **f** Western blot for PGC1B protein levels in *AAVS1*-CAG-CRISPRa hiPSCs harvested 4 days post-gRNA transduction. Experiments were performed in hiPSCs with dCas9-VPR transgene targeted to the *AAVS1* locus and CRISRPa-expressing hiPSCs via lentivirus (CRISPRa LV)

In efforts to generate a constitutive CRISPRa pluripotent stem cell line, the *AAVS1* safe harbor locus was targeted with dCas9-VPR driven by the CAG promoter in WTC11 hiPSCs (Figs. 1a, b, S1a). After selection, we identified several clones that correctly integrated the transgene at the desired location. Next, the expression of the transgene, dCas9-VPR, was measured at both the transcript and protein levels. To assess gene expression, transcript levels were assessed using qPCR (Fig. 1c). To eliminate PCR signal that may arise from potential contaminating genomic DNA, primers were designed to capture the post-transcriptional splicing event of the CAG promoter, flanking its intronic site (Fig. 1c). Of the 50 + clones screened, Clone 35.33 best expressed the mature, spliced CAG transcript at levels of 15% of the housekeeping gene, HPRT (Fig. 1c). This cell line generates stem cell colonies and maintains pluripotency (Fig. 1d). While transcripts were detected by qPCR, protein levels of dCas9-VPR were undetectable in these cells by western blot (Fig. S1b).

To test the functionality of the CRISPRa system, hiPSCs containing dCas9-VPR, were transduced with reporter GFP lentiviruses also containing guide RNA (gRNA) sequences (either control or designed for the GFP promoter) (Fig. S1c). All transduced cells weakly expressed GFP. However, if dCas9-VPR is present and active in the recipient cells, the cells receiving the lentiviruses with the gRNA targeting the GFP promoter (UbiC gRNA) should induce higher expression of GFP, in comparison to cells receiving lentivirus with control gRNA. Though expressing undetectable protein levels by western, *AAVS1*-CAG-dCas9-VPR Clone 35.33 (hereafter referred to as *AAVS1*-CAG-CRISPRa) hiPSCs show increased mean GFP fluorescence, indicative that minimal expression of dCas9-VPR protein is sufficient for functional activity (Fig. S1c). To test the efficacy in upregulating endogenous genes, *AAVS1*-CAG-CRISPRa hiPSCs were transduced with gRNAs for either *PPARGC1B* or *SOX6*, and non-targeting gRNAs were used as controls. *PPARGC1B* and *SOX6* genes have been identified as developmentally regulated transcription factors that control metabolism and electrical activity in cardiomyocytes [3, 23] and have low expression in hiPSCs and hiPSC-CMs, making them suitable targets for CRISPRa experiments. Transduction of gRNA enhanced mRNA expression of target genes an average of ~ 3.5 and ~ 100-fold respectively in comparison to non-targeting control samples (Fig. 1e). Upregulation of PGC1B (encoded by *PPARGC1B*) was readily demonstrated at the protein level as well (Fig. 1f). These trends are consistent with previous observations that levels of upregulation by CRISPRa are inversely proportional to basal expression levels [16, 34]. Comparing different clones, the levels of upregulation of target genes generally correlated with the levels of mature CAG transcript expression, however there are also gRNA functional differences across target genes and clones (genes more responsive than others in a clone-specific manner) (Fig. S1d). Thus, CRISPRa hiPSC clones were generated that upregulated target mRNAs in the pluripotent state. We focused subsequent studies on Clone 35.33 due to its high expression and strong induction of target gene expression (Fig. S1D).

### Cardiac differentiation of CRISPRa stem cells

**Fig. 2:**
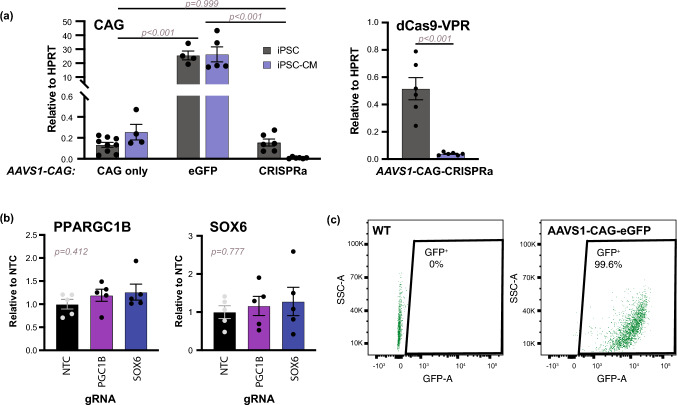
Cardiomyocyte differentiation of *AAVS1*-CAG-CRISPRa (Clone 35.33) hiPSC. **a** Gene expression analysis for mature CAG transcript is measured in hiPSCs and hiPSC-CMs (Day 14) for *AAVS1*-targeted cells (*left*). dCas9-VPR expression is shown in *AAVS1*-CAG-CRISPRa hiPSCs and after differentiation into hiPSC-CMs (*right*). Quantification relative to HPRT expression is shown. 2-way ANOVA, followed by post-hoc Tukey-Kramer test, (*cell line*: CAG only vs. CAG-eGFP vs. CAG-CRISPRa, p < 0.001; *cell state*: hiPSC vs. hiPSC-CM, p = 0.914; *cell line:cell state*: p = 0.980) and t-test were performed to determine statistical significance. *n = 4–9 (hiPSCs); n = 4–6 independent differentiations (hiPSC-CMs)*. **b** WTC11 *AAVS1*-CAG-CRISPRa hiPSC-CMs were transduced with gRNA (NTC, PPARGC1B, or SOX6) and harvested after 1 week for gene expression analysis using quantitative RT-PCR. Expression was normalized to HPRT and shown with respect to non-targeting control groups. One-way ANOVA was performed to calculate statistical significance. *n = 5 biological replicates.* **c** GFP expression in WTC11 *AAVS1*-CAG-eGFP hiPSC-CMs measured by flow cytometry. Wild type (WT) cells serve as negative gating controls. All error bars represent SEM

To test CRISPRa function in the differentiated state, *AAVS1*-CAG-CRISPRa hiPSCs were differentiated into hiPSC-CMs under adherent conditions using modulation of the Wnt pathway [9, 12]. *AAVS1*-CAG-CRISPRa cells successfully differentiated into enriched cardiomyocyte populations, indicated by spontaneous beating and cardiac troponin T positivity (Fig. S2a). When expression of the dCas9-VPR transgene was assessed by primers directly targeting the dCas9-VPR gene body or indirectly using the CAG intronic splicing event, expression was much lower than in the hiPSC state (< 1% of HPRT housekeeping gene in hiPSC-CMs compared to 15% in hiPSC for CAG; 4% in hiPSC-CMs vs. 51% in hiPSCs for dCas9-VPR) (Fig. 1c, 2a). To assess activity, day 14 hiPSC-CMs were assayed using the GFP reporter assay (Figs. S1c, S2b). *AAVS1*-CAG-CRISPRa hiPSC-CMs showed minimal upregulation of GFP expression. In contrast, transducing hiPSC-CMs randomly using lentivirus encoding dCas9-VPR resulted in robust upregulation of the GFP target gene (Fig. S2b). Moreover, when gRNAs targeting endogenous *PPARGC1B* and *SOX6* were introduced into the *AAVS1*-CAG-CRISPRa hiPSC-CMs, there was no impact on target gene expression (Fig. 2b). These results indicate that the dCas9-VPR transgene was not expressed after differentiation, and levels were insufficient to activate gRNA-targeted genes.

To assess expression activity at the *AAVS1* safe harbor site in hiPSCs, we switched the dCas9-VPR cassette for either eGFP (*AAVS1*-CAG-eGFP) or used the CAG promoter alone without a transgene (*AAVS1*-CAG only) (Fig. 1a). In undifferentiated hiPSCs, the *AAVS1*-CAG only cell line had comparable levels of transcription (13% of HPRT), in comparison to *AAVS1*-CAG-CRISPRa hiPSCs (Fig. S1d,  2a). Similarly, *AAVS1*-CAG-eGFP cells show strong uniform expression of GFP (Figs. S1e, f, 2a). Expression in these control lines was maintained after cardiomyocyte differentiation (Figs. 2a, c, S2a, c). These differences in mRNA levels of CAG mature transcripts are significantly influenced by cell line (two-way ANOVA, cell line: p < 0.001, differentiation state: p = 0.914), suggesting that silencing from the *AAVS1* safe harbor site is dependent on the cDNA sequence, not the promoter.

Taken together, these results demonstrate the low expression of the dCas9-VPR transgene in *AAVS1*-CAG-CRISPRa hiPSCs and further silencing during cardiac differentiation, while other cDNAs (eGFP) were readily expressed from the same promoter and locus [7, 38, 42].

### CRISPRa engineering of alternative safe harbor sites

**Fig. 3:**
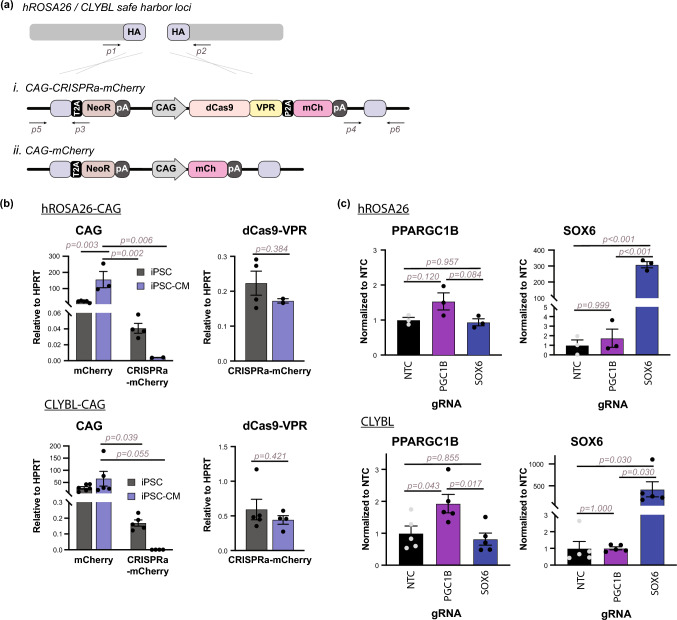
Targeting alternative safe harbor loci. **a** Strategy for CAG-CRISPRa-mCherry and CAG-mCherry cell lines targeting human *ROSA* (*hROSA26*) and *CLYBL* safe harbor sites in WTC11 stem cells. Double stranded breaks were generated at chr3:9396279–9396304 (between exons 1 and 2) for *hROSA26* and chr13:99822977–99822980 (between exons 2 and 3) for *CLYBL.* The following transgenic lines were generated: (*i*) dCas9-VPR-P2A-mCherry driven by the CAG promoter (*ii*) mCherry driven by CAG; all lines also contained neomycin resistance as a selection marker, with expression driven by the endogenous locus. **b** CAG transcript levels in hiPSCs and hiPSC-CMs (*left*). dCas9-VPR transcripts were further measured in CRISPRa-mCherry cells in undifferentiated and differentiated states (*right*). Expression is shown with respect to HPRT. 2-way ANOVA, followed by post-hoc Tukey-Kramer test, (*hROSA26 cell line*: CAG-mCherry vs. CAG-CRISPRa-mCherry, p = 0.007; *cell state*: hiPSC vs. hiPSC-CM, p = 0.004; *cell line:cell state*: p = 0.012; CLYBL *cell line*: CAG-mCherry vs. CAG-CRISPRa-mCherry, p = 0.011; *cell state*: hiPSC vs. hiPSC-CM, p = 0.202; *cell line:cell state*: p = 0.244) and t-test were performed to determine statistical significance. *n = 4–6 biological replicates (hiPSCs); n = 2–5 independent differentiations (hiPSC-CMs)*. **c** WTC11 hiPSCs were transduced with indicated gRNAs and harvested for gene expression analysis. mRNA expression is normalized to HPRT housekeeping gene and shown with respect to non-targeting control gRNA samples. One-way ANOVA, followed by post-hoc Tukey-Kramer test, was performed to calculate statistical significance. *n = 3 (hROSA26) or 5 (CLYBL) biological replicates.* All error bars represent SEM

To test the hypothesis that silencing of dCas9-VPR was unique to the *AAVS1* locus, we generated WTC11 stem cell lines targeting mCherry or dCas9-VPR-P2A-mCherry to both the human *ROSA26* (*hROSA26*) [30] and *CLYBL* [15] safe harbor loci (Figs. 3a, S3a). The P2A serves as a post-translational self-cleaving peptide to generate dCas9-VPR and mCherry peptides [37], allowing for monitoring of transgene expression via mCherry fluorescence. The *hROSA26* locus was previously demonstrated to give rise to high expression of an eGFP reporter in human embryonic stem cells and to remain active after differentiation [7]. In parallel, we targeted the *CLYBL* safe harbor locus, which was used more recently for the generation of constitutive CRISPRi and CRISPRa WTC11 cell lines [59, 60]. To better track transgene expression, we incorporated mCherry into our CRISPRa targeting constructs to generate *hROSA26*-CAG-CRISPRa-mCherry and *CLYBL*-CAG-CRISPRa-mCherry cell lines (Clones 4 − 1 and 2, respectively) (Figs. 3a, S3a). During pluripotency (Fig. 3b), the mCherry-only cell lines, *hROSA26*-CAG-mCherry (Clone 7) and *CLYBL*-CAG-mCherry, had 500-fold (*hROSA26*) and 170-fold (*CLYBL*) greater levels of CAG transcripts in comparison to CRISPRa-mCherry lines. Studying protein expression by flow cytometry, only 13% of the *hROSA26*-CAG-CRISPRa-mCherry line were mCherry-positive, while the *hROSA26*-CAG-mCherry cells were uniformly, strongly positive (Fig. S3c). Moreover, *CLYBL*-CAG-CRISPRa-mCherry lines weakly express mCherry in hiPSCs (41% positive) while the *CLYBL*-CAG-mCherry hiPSCs strongly express mCherry (Fig. S3c). It is important to note that the expression in the CAG-mCherry hiPSCs and hiPSC-CMs was incredibly strong, and the voltage settings were dialed down to accommodate visualization of cells on plots. Though both the *hROSA26* and *CLYBL* CAG-CRISPRa-mCherry stem cells showed weak expression, these cells upregulated native *PPARCG1B* and *SOX6* gene expression after gRNA transduction to levels comparable to the *AAVS1*-targeted cells (Fig. 3c; compare to Fig. 1e).

These alternatively targeted stem cells also underwent successful differentiation into cardiomyocytes (Fig. S3b). While *hROSA26* and *CLYBL* CAG-mCherry control lines maintained robust expression, both lines of CAG-CRISPRa-mCherry hiPSC-CMs lost transgene expression, measured by RT-PCR (< 1% of HPRT in hiPSC-CMs) and flow cytometry, indicating silencing during differentiation (Figs. 3b, S3d). These results indicate that the silencing of dCas9-VPR expression is independent of the targeted human safe harbor site.

### Regulation of CRISPRa mediated by muscle regulatory cassettes

**Fig. 4:**
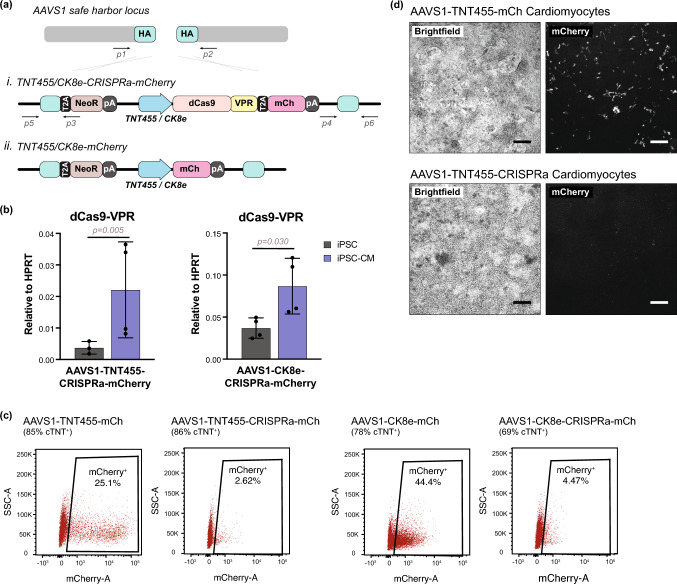
Utilization of muscle-specific promoters to drive transgene expression in cardiomyocytes. **a** Schematic of targeting constructs introduced to the *AAVS1* safe harbor locus between exons 1 and 2, driven by muscle regulatory cassettes. The following transgenic lines were generated: (*i*) dCas9-VPR-P2A-mCherry driven by the TNT455 or CK8e promoter (*ii*) mCherry driven by TNT455 or CK8e promoter; all lines also contained neomycin resistance as a selection marker, with expression driven by the endogenous locus. **b** dCas9-VPR RNA expression in hiPSCs and hiPSC-CMs (Day 14) in *AAVS1*-targeted cell lines. Gene expression is normalized to HPRT housekeeping gene. A t-test was performed to determine statistical significance. *n = 3–4 biological replicates (hiPSCs); n = 4 independent differentiations (hiPSC-CMs)*. Error bars represent SEM. **c** mCherry expression analysis in Day 14 hiPSC-CMs by flow cytometry. Gating was determined based on age-matched unedited wild type WTC11 hiPSC-CMs. Percent cTNT positive cells are indicated for each differentiation. **d** mCherry expression from TNT455-regulated cassettes with and without the inclusion of dCas9-VPR as part of the cDNA in Day 14 WTC11 hiPSC-CMs. *Scale bar: 200 μm*

To test if silencing of transgene expression is dependent on promoter regulation during phases of differentiation, the CAG promoter was replaced with cell type-specific promoters, either the striated muscle-specific creatine kinase (CK8e) [27] or cardiac-specific troponin T (cTNT) regulatory cassettes (TNT455) [33]; these constructs were introduced to the *AAVS1* safe harbor site (Figs. 4a, S4a). The CK8e regulatory cassette is a miniaturized and control-element-altered derivative of the endogenous mouse creatine kinase promoter, one of the most highly expressed genes in skeletal muscle and cardiac muscle [51]. The TNT455 cassette is modified from the endogenous human cTNT promoter, containing a miniaturized set of essential control elements [33]. Pilot studies demonstrated that the TNT455 promoter has ~ 2.5-fold greater activity than CK8e in WTC11 hiPSC-CMs (not shown). After performing the knock-ins and qualifying the clonal lines, the hiPSCs were differentiated into cardiomyocytes. To our surprise, these *AAVS1*-CK8e-CRISPRa-mCherry (Clone 7) and *AAVS1*-TNT455-CRISPRa-mCherry (Clone 3–11) hiPSC-CMs showed only a slight induction of dCas9-VPR transcript (2–8% of HPRT, comparable to levels in hiPSC-CMs of CAG-driven expression (Fig. 2a)) and virtually no visible expression of mCherry (Fig. 4b–d). Control lines lacking the dCas9-VPR cDNA component (*AAVS1*-CK8e-mCherry and *AAVS1*-TNT455-mCherry) demonstrated expression of the fluorescent reporter at day 12 of differentiation (Fig. 4c, d). Cardiomyocytes were also transduced with gRNA lentivirus for endogenous genes, however induced mRNA expression levels of target genes, *PPARGC1B* and *SOX6*, were not sufficient to show evidence of activity in hiPSC-CMs (Fig S4B).

To assess whether promoter activity is in part regulated by hiPSC-CM maturation state, hiPSC-CMs underwent a maturation culture protocol for an additional week using low glucose medium, supplemented with dexamethasone and thyroid hormones (conditions previously demonstrated to promote the maturation of cardiomyocytes in vitro) [40, 48, 63]. The intensity of mCherry in expressing populations increased in more mature *AAVS1*-CK8e-mCherry hiPSC-CMs (Fig. S4c), suggesting the activity of the CK8e regulatory cassette is in part influenced by the maturation state of the cell. This is in line with the effects of maturation medium on the expression of endogenous cardiac troponin T and muscle creatine kinase. In contrast, the maturation medium did not activate expression from the muscle regulatory cassettes when dCas9-VPR-P2A-mCherry was the cDNA.

### Silencing in endothelial cells

**Fig. 5:**
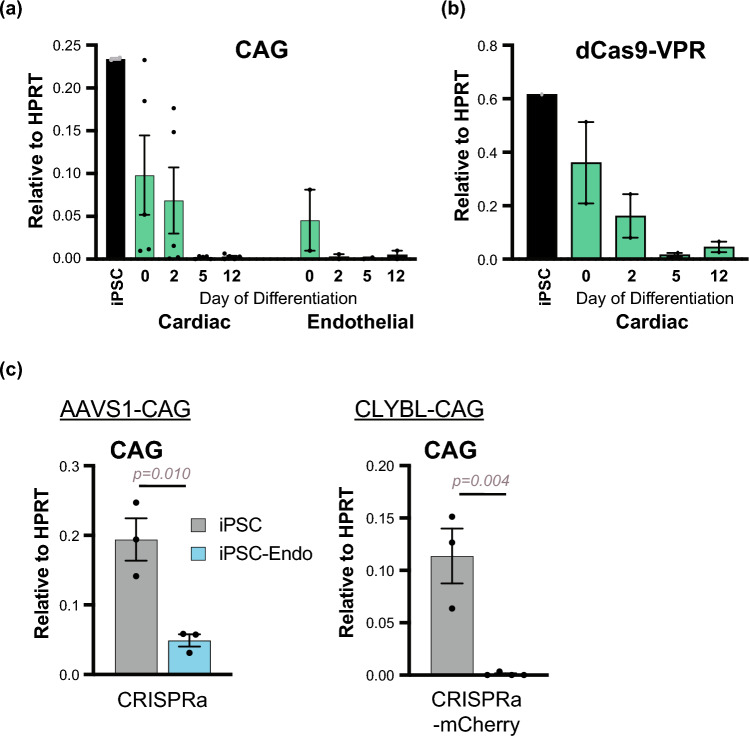
Differentiation from mesoderm lineage. WTC11 *AAVS1*-CAG-CRISPRa hiPSCs were differentiated into hiPSC-CMs and cardiogenic endothelial cells (iPSC-Endo). Cells were harvested across different time points during differentiation and mature CAG transcript expression was measured. Days of differentiations are indicated (Day 0- primed stem cell; Day 2- mesoderm; Day 5- progenitor; Day 12- cardiomyocyte or endothelial cell). mRNA expression of transcript measured at CAG promoter (**a**) and at dCas9-VPR (**b**) is normalized to HPRT. **c** CAG-mediated expression measured in *AAVS1*-CAG-CRISPRa and *CLYBL*-CAG-CRISPRa-mCherry cell lines in undifferentiated and Day 14 hiPSC-Endo. A t-test was performed to determine statistical significance. *n = 3 biological replicates (hiPSCs); n = 2–4 independent differentiations (hiPSC-CMs); n = 3–4 independent differentiations (hiPSC-Endos)*. All error bars represent SEM

To assess when this silencing occurs during the scope of differentiation, *AAVS1*-CAG-CRISPRa cells were harvested at different stages of the cardiomyocyte differentiation protocol: day 0 (hiPSC), day 2 (mesoderm), day 5 (cardiac progenitor) and day 12 (cardiomyocyte) (Fig. 5a, b). Results show a sharp decline in expression of both the CAG and the dCas9-VPR transcripts between days 2 and 5. These results are consistent with other reports that show detectable dCas9-VPR transgene expression at the mesoderm stage with their validations of differentiation [22, 24, 53]. However, these studies do not report on expression at later stages. To test whether these effects are specific to the cardiomyocyte lineage post-mesoderm (Day 2), we performed a parallel differentiation protocol to generate cardiac endothelial cells (hiPSC-Endo) as previously described [47]. Consistent with the cardiomyocyte data, endothelial progenitors also silenced the CAG promoter during differentiation (Fig. 5a). These results are consistent across both *AAVS1* and *CLYBL* targeted CRISPRa cell lines (Fig. 5c).

### DNA methylation of the CAG promoter

**Fig. 6:**
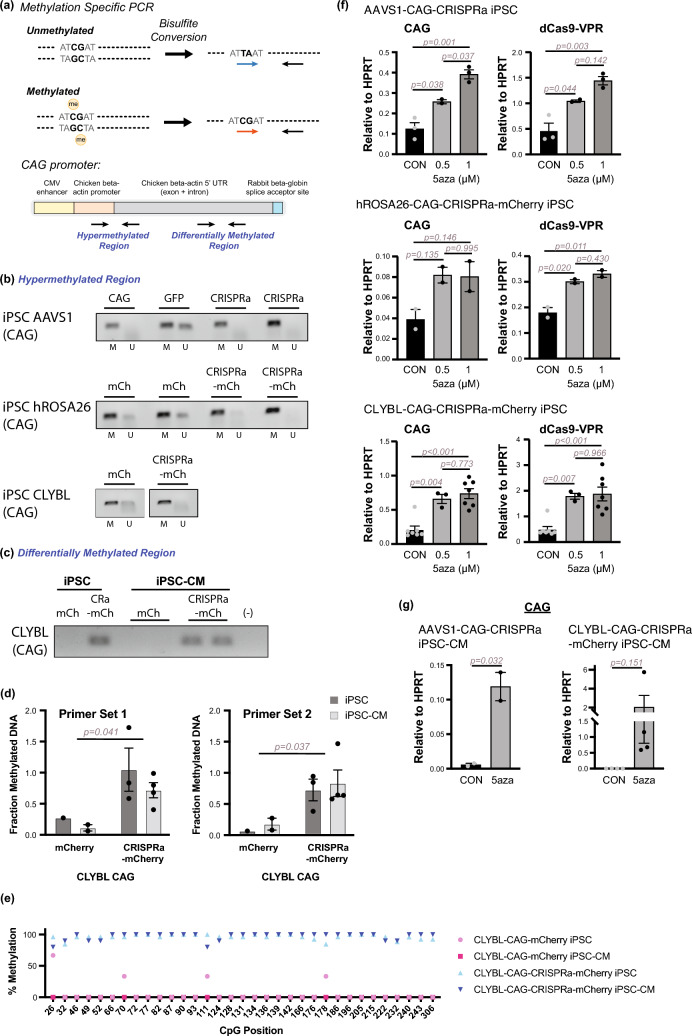
DNA methylation analysis of promoters. **a** Schematic of methylation-specific PCR (MSP) assay. Genomic DNA is subjected to bisulfite conversion. Converted DNA is used as input for PCR with primers specific for either methylated or unmethylated cytosines for the same site, and subsequently assessed for the presence or absence of product indicating the methylation status of the target region. Locations of MSP primer target sites measured for the CAG promoter are indicated. **b** WTC11 hiPSC lines, with genes driven by CAG that had been targeted to either the *AAVS1*, *hROSA26*, or *CLYBL* genomic loci, were subjected to bisulfite conversion, followed by PCR using primers targeting methylated (M) or unmethylated (U) DNA. Representative gel images are shown for analysis of the hypermethylated region of the CAG promoter. **c** MSP analysis of differentially methylated region of CAG promoter, using primers targeting methylated DNA, in *CLYBL*-targeted cell lines. **d** Genomic DNA from hiPSCs and hiPSC-CMs (Day 14) in *CLYBL*-targeted cell lines were subjected to methylation sensitive HpaII digestion. Quantitative PCR measures fraction of methylated DNA in the intronic region of the promoter. *n = 1–4 biological replicates.* 2-way ANOVA, followed by post-hoc Tukey-Kramer test, (Primer Set 1: *CLYBL*-CAG-mCherry vs. *CLYBL*-CAG-CRISPRa-mCherry, p = 0.0413; *cell state*: hiPSC vs. hiPSC-CM, p = 0.304; *cell line:cell state*: p = 0.772; Primer Set 2: *CLYBL*-CAG-mCherry vs. *CLYBL*-CAG-CRISPRa-mCherry, p = 0.037; *cell state*: hiPSC vs. hiPSC-CM, p = 0.650; *cell line:cell state*: p = 0.996). **e** Bisulfite PCR was performed to amplify the CAG intronic sequence. Sanger sequencing was used to assess and quantify percent methylation at individual CG sites within amplicon, spanning 33 CpGs. Plot shows percent methylation of CpGs in sampled PCR products from CLYBL cell lines. x-axis indicates CpG position within the PCR amplicon. **f** hiPSCs were treated with indicated concentrations of 5-azacytidine (5aza) for 24 h and harvested for mRNA expression analysis of CAG or dCas9-VPR by quantitative rt-PCR. Expression is quantified with respect to HPRT. One-way ANOVA, followed by post-hoc Tukey-Kramer test, was performed to calculate statistical significance. *n = 2–7 biological replicates*. **g** Day 16 hiPSC-CMs were treated with 5µM 5aza for 96 h and harvested for CAG transcript expression analysis by quantitative rt-PCR. Expression is quantified with respect to HPRT. A t-test was performed to determine statistical significance. All error bars represent SEM

The results above indicate that regardless of safe harbor or promoter, dCas9-VPR is silenced to non-functional levels in hiPSC-CMs, while fluorescent protein reporters are active with the same promoters and loci of integration. Previous reports have demonstrated that the CAG promoter can be subjected to DNA methylation [44, 64, 65]. To assess DNA methylation, genomic DNA isolated from stem cells was subjected to bisulfite treatment and analyzed using methylation specific PCR (MSP) (Fig. 6a). MSP analysis of the hypermethylated region [64] of the CAG promoter shows the presence of a strong band, indicating methylation at the CAG promoter across all of the CRISPRa hiPSC lines irrespective of the *AAVS1*, *hROSA26*, or *CLYBL* genomic loci to which the transgenes were targeted (Fig. 6b). Interestingly the control fluorescent protein cell lines (*AAVS1*-CAG-eGFP, *hROSA26*-CAG-mCherry and *CLYBL*-CAG-mCherry) show the presence of a band for unmethylated DNA. These patterns correlate with the relative CAG transcript expression patterns in hiPSCs measured across cell lines (Figs. 2a and 3b). Next, methylation was assessed within the differentially methylated region, annotated within the intronic region. Using MSP primers targeting methylated DNA, *CLYBL*-CAG-CRISPRa-mCherry lines exhibited methylation in both hiPSC and hiPSC-CM states (Fig. 6c). To quantify methylation, genomic DNA was digested with HpaII, a methylation sensitive enzyme unable to cut methylated CCGG motifs, and then amplified using qPCR. *CLYBL*-CAG-CRISPRa-mCherry cells, hiPSCs and hiPSC-CMs, had a higher fraction of methylated DNA in comparison to *CLYBL*-CAG-mCherry lines (Fig. 6d), consistent with patterns of transcript expression. To further assess promoter methylation on a broader scale, bisulfite-treated DNA was amplified using primers targeting previously identified methylation-regulated region of the CAG promoter, spanning the chicken beta-actin intron region [64]. Consistent with previous results, bisulfite sequencing analysis of *CLYBL*-CAG-mCherry and *CLYBL*-CAG-CRISPRa-mCherry hiPSCs and hiPSC-CMs demonstrates saturated levels of CpG methylation for this region of the CAG promoter in CRISPRa vs. mCherry cell lines (Fig. 6e). Additionally, hiPSCs and hiPSC-CMs were treated with 5-azacytidine, an inhibitor for DNA-methyltransferase. After 24 h, concentration-dependent increases in transgene expression were observed in hiPSCs (Fig. 6f). These patterns were consistent in hiPSC-CMs after 4 days 5-azacytidine treatment, demonstrating increases in transcript abundance after inhibition of DNA-methyltransferase activity (Fig. 6g). These results suggest that the relatively low levels of expression arising from the CAG promoter in undifferentiated pluripotent stem cells and hiPSC-CMs may be attributed to DNA methylation.

## Discussion

In this study, we sought to generate a constitutively active CRISPRa stem cell line. In these efforts, we screened stem cell lines, generated via different strategies, with multiple clones and subclones for each. While the dCas9-VPR, driven by the CAG promoter, was active in the undifferentiated state, the expression and activity from this transgene was lost after differentiation to cardiomyocytes or endothelium. Silencing occurred in multiple safe harbor loci and under control of both ubiquitous and muscle-specific promoters. Interestingly, genes encoding fluorescent reporter proteins continued to be strongly expressed after differentiation under all conditions, pointing to the dCas9-VPR cDNA as the driver of silencing. Finally, we provide evidence that DNA methylation of the promoter correlates with weak expression of CRISPRa transgene, compared to control fluorescent reporter lines. These findings demonstrate significant challenges in engineering a CRISPRa hiPSC for cardiomyocyte or endothelial applications. Moreover, this highlights that cell lines should be carefully tested for each application, the need to identify a permissible safe harbor site, and the importance of identifying appropriate promoters with optimal activity in the cell type of interest.

The weak expression and silencing observed of the CRISPRa transgene is consistent across safe harbors and promoters utilized. Why are differential levels of expression arising from the same targeting design observed when combined with different cDNAs (fluorescent protein vs. dCas9-VPR)? One possibility is the VPR sequence itself and its viral origins; i.e. VP64 and/or the Rta viral components within the synthetic VPR sequence may mediate cellular DNA methylation defense mechanisms. As a part of their innate immune response, cells have developed means of recognizing and silencing foreign viral DNA through epigenetic mechanisms [61]. If such response is taking place, further rounds of codon optimization of sequence, accommodating host translational machinery, may reduce DNA foreignness and combat this epigenetic mechanism of silencing. Interrogation of dCas9-VPR components to assess if a given region of the transgene is driving silencing can provide insights into mechanism for CAG methylation, and perhaps methylation of other promoters. Alternatively or additionally, the size of the gene insert may play a role. The dCas9-VPR gene is roughly 6 kb vs. the 700 bp size of eGFP or mCherry. The large insert may be disrupting the local chromatin landscape, causing the locus to shut down. It was recently described that the human silencing hub (HUSH) complex serves as a eukaryotic defense mechanism to silence long foreign intronless DNA introduced to cells [55]; this mechanism can contribute to shutting off dCas9-VPR expression, which does not include intronic sequences. Perhaps, incorporating additional introns (the CAG promoter already contains an intronic sequence for enhanced expression) or exploring the CRISPRa split systems, including SunTag [58] and SAM [34], to introduce as fragments and thereby minimize insert sizes, can be interrogated in future studies. During the time of our experiments, the same dCas9-VPR sequence at the AAVS1 locus was demonstrated to be functional in iPSC-CM. This study did not report whether differences in expression were observed in undifferentiated vs. differentiated states, however authors demonstrate substantial increases in dCas9-VPR protein expression in hiPSCs and activity in hiPSC-CMs when a WPRE sequence, previously demonstrated to boost retroviral transgene expression, tagged their construct [54, 66].

The regulation of the CAG promoter is dependent on its downstream sequence. In both the hiPSC and cardiomyocyte states, differential DNA methylation patterns are observed between mCherry and dCas9-VPR-P2A-mCherry lines. While we do not observe changes between hiPSC vs. hiPSC-CM in the “differentially methylated” portion of the promoter, the methylation differences across cell lines (CAG-mCherry vs. CAG-CRISPRa) may explain the differences in global expression. The further silencing measured in CRISPRa hiPSC-CM after differentiation may be attributed to other epigenetic factors not measured in this study, and the upregulation of CRISPRa gene expression in hiPSC-CMs after 5-azacytidine treatment (Fi﻿g. 6g) implicates methylation in regions not tested in our PCR screen. Interestingly, applying muscle-specific promoters did not improve expression. The CK8e and TNT455 promoters have been designed for optimal activity in adult muscle cells. The hiPSC-CMs are not mature [31], hence the cells may not express the components and regulatory proteins needed to optimally activate the regulatory cassettes. Driving hiPSC-CM maturation via low glucose and metabolic hormone treatment, while effective in enhancing mCherry expression from the CK8e promoter, was not sufficient to induce dCas9-VPR expression in cells, suggesting additional regulatory components are necessary. While an inducible promoter has been used for dCas9-VPR targeted to the *AAVS1* safe harbor locus [22, 24], it is important to note that these groups studied transgene induction only in the undifferentiated state. In the case of CRISPR and CRISPR interference, it has been pointed out that the inducible system may also be subjected to silencing in iPSC-CM [7, 39].

These lessons reported here can be applied to broader contexts, such as gene therapy and cellular reprogramming, in which long term expression of transgenes is essential for therapeutic benefits or maintenance of cell identity [2, 43]. The observation of silencing is not limited to the CAG promoter. Comparisons of different cell type-specific promoters in the context of myeloid differentiation demonstrate varying activity, associated with promoter methylation [32]. Similar to our study, silencing of viral promoters has been observed in dividing cells and across stem cell differentiation, attributed to promoter methylation [19, 25]. The EF1$\alpha$ promoter more recently used to generate transgenic mice demonstrated inactivity and hypermethylation [6]. Perhaps, a contributing feature is the species mismatch between promoter utilized and host cell, leading to a defense response. While there are broad examples of transgene silencing, the commonalities in mechanism has yet to be teased out in different scenarios, whether this silencing occurs immediately post-integration, is reflective of variegation across cells derived from a clone or is a function of proliferation and differentiation [19]. In the studies applying lentiviral transduction where multiple instances of integration may occur within a given cells, cases of silencing can be often masked and overlooked, as selection of a successful clone is only dependent on expression from one integration site. Here, we aimed, when possible, to focus on clones with homozygous or heterozygous genotype to correlate findings directly to targeted loci.

Several limitations of this study should be pointed out. We focused on three well-utilized human safe harbor sites; however, it is possible that better universal safe harbor sites, that are on during pluripotency and maintained with differentiation, may yet be determined. Recently, a panel of human safe harbor sites were identified and were shown to have high activity in human cells and to support dCas9-VPR expression [49]. It will be important to test expression and functional dynamics at these safe harbor sites across cell types and during development/differentiation. Additionally, we evaluated the promoter activity and transgene expression in the WTC11 cell line and the findings are limited to this genetic context. Our study is not the first to describe transgene silencing [13], or silencing of the *AAVS1* locus with stem cell differentiation [7, 10, 32, 45], and this observation may be due to promoter-dependent and differential DNA methylation effects [32] that change during hiPSC maturation. While our results suggest that silencing is a promoter-independent feature of the dCas9-VPR cassette that is shared across different safe harbor sites, we did not examine alternative CRISPRa systems or transgenes other than mCherry and GFP reporters. We also did not attempt targeted CRISPR-mediated epigenomic DNA modifications that have the potential of activating gene expression without using VPR strategies [62]. In our studies transgene silencing occurred with both the constitutively active CAG promoter and with muscle-specific promoters derived from the muscle creatine kinase and cardiac troponin T genes; however, complementary interrogation of whether other constitutive promoters, such as EF1$\alpha$, or inducible promoters also experience activity loss over the course of differentiation is important. Although our studies showed that DNA methylation of the CAG promoter and that overall cell line methylation was greater when it was ligated to dCas9-VPR than to mCherry cDNA, other epigenetic mechanisms active during differentiation, including histone marks, may be at play also [4]. Finally, we examined only cardiomyocyte and endothelial cell types derived from a mesoderm lineage, and did not consider endodermal or ectodermal cell lineages. Lessons from this study can be taken into consideration more broadly for general genomic engineering strategies.

## Methods

### Stem cell culture and differentiation

WTC11 human induced pluripotent stem cells (gifted from Dr. Bruce Conklin, Gladstone Institute; available from Coriell #GM25256) were maintained in culture using mTeSR medium (Stem Cell Technologies). Cardiomyocyte differentiation was performed as previously described using small molecules [9]. Stem cells were plated in 24-well plates, pre-coated with Matrigel (Corning), in mTeSR supplemented with 10µM Y-27632 (Stem Cell Technologies). The next day (day − 1), cells were primed with 1µM CHIR-99021 (Cayman). On day 0, medium was switched to RPMI-1640 + 500 µg/ml BSA + 213 µg/ml ascorbic acid (RBA) supplemented with 3–5µM CHIR-99021 to induce mesoderm formation via Wnt activation, followed by Wnt inhibition at Day 2 to drive cardiomyocyte differentiation (2µM Wnt-C59, Biogems). On day 4, medium was changed to RBA only. From day 6 and every other day onwards, cardiomyocytes were maintained in RPMI-1640 + 1X B27 (plus insulin) (Life Technologies) until harvested at time points (Day 14 or as indicated). Endothelial cell differentiation was performed as previously described [47]. Stem cells were plated in 24-well plates in mTeSR + 10µM Y-27632 + 1µM CHIR-99021. After 24 h (day 0), medium was changed to RPMI-1640 + 1X B27 (minus insulin) + 100ng/ml activin A (R&D) + 1X Matrigel. On day 1, cells were fed with RPMI-1640 + 1X B27 (minus insulin) + 5ng/ml BMP4 (R&D) + 1µM CHIR-99021. The next day, medium was changed to StemPro-34 (Life Technologies) + 0.4mM 1-thioglycerol (Sigma) + 50 µg/ml ascorbic acid + 10ng/ml BMP4 + 5ng/ml bFGF (Peprotech) + 200ng/ml VEGF (Peprotech) + 2mM L-glutamine. After 72 h, cells were replated on gelatin-coated flasks and maintained in EGM-2 (Lonza) + 20ng/ml bFGF + 20ng/ml VEGF + 1µM CHIR-99021 until harvested.

### CRISPRa cloning/targeting strategies

To generate the *AAVS1* donor plasmids in this study, we used the pAAV-Neo-CAG donor backbone, containing a cassette expressing a gene-trap driven neomycin resistance gene followed by a CAG promoter, and flanked by *AAVS1* homology arms. For the AAVS1-CAG-dCas9-VPR plasmid, dCas9-VPR (a gift from Kristen Brennand, Addgene #99373 [28]) was PCR amplified and cloned into the pAAV-Neo-CAG plasmid. For AAVS1-CAG-eGFP generation, AAV-CAGGS-EGFP (a gift from Rudolf Jaenisch, Addgene #22212 [29]) donor plasmid was used. In CAG-only control cells, the pAAV-Neo-CAG was directly introduced to hiPSCs (no additional cloning). Striated muscle-specific CK8e and TNT455 promoter constructs were provided by Dr. Stephen Hauschka [27, 33]. To generate the donor CRISPR-mCherry plasmids driven by muscle regulatory cassettes, the promoters were assembled with dCas9-VPR-P2A (Addgene #99373) and mCherry (a gift from Jacob Corn, Addgene #102245) and cloned into the pAAV-Neo-CAG donor backbone, replacing the CAG fragment (pAAV-[CK8e or TNT455]-dCas9-VPR-P2A-mCherry). The mCherry control plasmids were generated assembling the promoter and mCherry fragments (pAAV-[CK8e or TNT455]-mCherry). For generation of the *hROSA26* and *CLYBL* safe harbor donor plasmids, dCas9-VPR-P2A (Addgene #99373) and mCherry (Addgene #102245), or mCherry only, were assembled into *hROSA26* CAG-eGFP [7] or pC13N-iCAG.copGFP (a gift from Jizhong Zou, Addgene #66578 [15]) plasmids, respectively, replacing the GFP region. PCR of fragments were performed using Q5 High Fidelity DNA Polymerase (New England Biolabs). All cloning reactions were performed using NEBuilder HiFi DNA Assembly mix (New England Biolabs) at a 2:1 molar ratio of insert:backbone. Plasmid cloning was confirmed via Sanger sequencing and restriction digestion prior to cell transfection.

To target the safe harbor sites, the following targeting plasmids were co-transfected with donor plasmids: *AAVS1* locus- pZFN_AAVS1-R-KKR and pZFN_AAVS1-L-ELD [7, 8] to induce break between exons 1 and 2 of *AAVS1* locus; *CLYBL*- pZT-C13-R1 and pZT-C13-L1 (a gift from Jizhong Zou, Addgene #62196, #62197 [15]) to induce break between exons 2 and 3 of *CLYBL* locus; *hROSA26*- pSpCas9n(BB)_R26-L and pSpCas9n(BB)_R26-R [7] to induce break between exons 1 and 2 of *hROSA26* locus. WTC11 human induced pluripotent stem cells were co-transfected with 2 µg total of targeting plasmids and donor plasmid (equal mass for each plasmid) using GeneJuice Transfection Reagent (Millipore Sigma). After transfection, cells were selected with 50 µg/ml G418 for 3 days, then maintained in 25 µg/ml G418 for an additional week. Following selection, single cells were replated and individual clones were picked for genotyping and expansion.

### Genotyping

Genomic DNA was isolated using DNeasy Blood and Tissue kit (Qiagen). PCR was performed using LongAmp Taq polymerase (New England Biolabs) and primers designed for assessment of safe harbor site integrity, transgene integration, and random integration. PCR products were run on 0.8% agarose gel and assessed for presence of bands. Primer targeting sites are indicated in Figs. 1a and 3a. Refer to Supplemental Table 1 for primer sequences.

### Cardiomyocyte maturation

To select for mature hiPSC-CMs, cardiomyocytes were fed every two days with RPMI-1640 (no glucose) (Life Technologies) supplemented with maturation medium (1mM dextrose [Sigma] + 1µM Dexamethasone [Sigma] + 100nM T3 [Sigma]). Cells were harvested after 1 week for analysis.

### gRNA design

CRISPRa guide RNAs for targeting gene promoter region (-300 to 0 bp of transcription start site) were designed using Genetic Perturbation Platform sgRNA Design tool (Broad Institute). gRNA sequences (IDT) were cloned into the CROPseq opti backbone (a gift from Jay Shendure, Addgene #106280 [26]). For each target gene, 2–3 gRNA plasmids were pooled. Lentiviruses were generated from the plasmid pool and titered by the Fred Hutch Viral Vector core. Non-targeting control gRNA sequences were selected from a list of human non-targeting guides (Sanjana Lab). Refer to Supplemental Table 2 for gRNA sequences. To test CRISPRa-mediated upregulation of endogenous genes, cells were transduced at an MOI 0.5 followed by puromycin selection (hiPSC: 1$\upmu$g/ml for 3 consecutive days; hiPSC-CM: 2$\upmu$g/ml for 4 consecutive days). Cells were harvested 4 days or 7 days post-transduction for hiPSCs and hiPSC-CMs, respectively, for downstream analysis.

### Quantitative PCR

RNA was isolated from whole cell lysate using RNA Miniprep kit (Zymo). cDNA was synthesized using random primers by MMLV reverse transcriptase (Life Technologies) with 500ng input RNA. Quantitative real-time PCR was performed using SYBR green master mix (Life Technologies). HPRT was used as a housekeeping gene. Refer to Supplemental Table 3 for primer sequences.

### Western blot analysis

Cell pellets were lysed on ice using RIPA lysis buffer (Life Technologies). Whole cell protein concentration was quantified using the BCA assay (Pierce). Proteins samples were diluted with 4X Laemmli buffer (Biorad). 30 µg of protein was loaded to 4–20% gradient gel (Biorad). After gel electrophoresis, protein transfer to PVDF membrane (Millipore) was performed. Membrane was incubated in blocking buffer (5% non-fat dry milk in PBS + 0.1% Tween (PBST)), followed by overnight incubation with primary antibody in blocking buffer at 4 C (1:1000 antibody dilution). Membrane was washed with PBST, then incubated with secondary antibody (1:10,000 dilution) at room temperature for 2 h. Following PBST washes, the membrane was incubated with SuperSignal West Pico chemiluminescent substrate (Life Technologies) and imaged with Biorad gel-doc. The membranes were stripped with Restore Western Blot Stripping buffer, washed, blocked, and re-probed with antibody to assess expression of loading controls. Supplemental Table 4 lists antibodies used.

### Flow cytometry

Cells were harvested, fixed with 4% paraformaldehyde at room temperature, 10 min. Afterwards cells were washed and stained using antibodies for pluripotency or cardiac markers (OCT4 or cardiac troponin T), diluted in 0.75% saponin in 5% FBS/PBS, for 1 h at room temperature. After incubation, cells were washed, and fixed samples were run on CantoII (BD).

To assess mCherry or eGFP expression, hiPSCs or hiPSC-CMs were harvested with versene (Gibco) or 0.25% trypsin solution (Gibco), respectively, washed with PBS, and stained with 0.2$\upmu$g/ml DAPI for exclusion of dead cells. Cell suspension was run through LSRII flow cytometer (BD) to assess the mCherry or eGFP positive cell populations (percent positive and intensity). Wild type cells were used for gating. Due to strong expression of mCherry/eGFP in control cell lines (CAG-eGFP and CAG-mCherry), the voltage settings were lowered for these samples to center cells on flow plots.

### CRISPRa activity assays

CRISPRa activity was assessed using CRISPRaTest Functional dCas9-Activiator Assay Kit (Cellecta) according to manufacturer instructions. CRISPRa hiPSCs or hiPSC-CMs were replated in 12-well plates, 3 wells per cell line/state (Day 0). For each dCas9-VPR containing cell line, one well served as a negative control (untransduced), one well received background control lentivirus (negative control expressing non-targeting gRNA), and one well received active lentivirus (expressing gRNA for the Ubiquitin C promoter). Medium was changed on days 1, 2, and 3. Cells were harvested on Day 4, and GFP intensity was measured by running flow cytometry using a CantoRUO instrument (BD). All CRISPRa cells receiving lentivirus (either control or Ubiquitin C promoter gRNA) should weakly express GFP. If cells express sufficient levels of dCas9-VPR and received the lentivirus containing a gRNA for the Ubiquitin C promoter, the GFP reporter should be further activated, enhancing GFP fluorescence intensity detected (Fig. S1c).

### DNA methylation assays

Genomic DNA was isolated using DNeasy Blood and Tissue kit (Qiagen), treated with RNase A (Zymo) and used as input for bisulfite conversion using an EZ DNA Methylation kit (Zymo). Methylation Specific (MSP) PCR primer sets were designed using MethPrimer [36] or previously published [64], and used to assess promoter methylation using Hot Start Taq polymerase (New England Biolabs). Refer to Supplemental Table 5 for primer sequences. For DNA methylation inhibition experiments, stem cells were treated with 5-azacytidine (Stem Cell Technologies) for 24 h and harvested for RNA to assess CAG transcript expression. For hiPSC-CM analysis, cells were treated with 5 µM 5-azacytidine at days 16 and 18 of differentiation and harvested at day 20 for RNA analysis (96 h post-initiation of treatment).

HpaII digestion was performed to quantify DNA methylation within the differentially methylated region of the CAG promoter. Isolated genomic DNA (500 ng) from indicated samples was digested with methylation sensitive HpaII enzyme (New England Biolabs) for 45 min at 37 C. Following digestion, enzyme was inactivated. Purified DNA was used as input for quantitative PCR to measure uncut product, corresponding to methylated DNA. Analysis was performed by first normalizing values to control genomic regions (lacking CCGG recognition site), followed by normalization to undigested DNA to assess fraction of methylated DNA.

For Bisulfite sequencing analysis, the differentially regulated region of the intronic region of the CAG promoter was amplified using previously published primers, with coverage of 33 CpGs [44]. PCR products were cloned into pGEM-T Vector (Promega) following manufacturer’s protocol, transformed into JM109 Competent Cells and grown on LB agar plates containing 50$\upmu$g/ml Ampicillin + 100mM IPTG (ThermoFisher) + 20 mg/ml X-Gal (ThermoFisher) overnight at 37 C. The following day, individual white colonies were selected and further cultured in LB broth. Purification of plasmid was performed using a Miniprep kit (Qiagen). Plasmids were submitted for Sanger sequencing of PCR product insert. Sequences were analyzed using QUMA web-based tool to assess and quantify CpG methylation at individual sites [35].

### Statistics

Statistical analysis was performed using R (version 4.1.2) for the indicated statistical tests to assess significance of results.

### Supplementary Information

Below is the link to the electronic supplementary material.

Supplementary material 1 (DOCX 16515 kb)

## Data Availability

Data and materials generated and used in this study are available upon request.

## Declarations

### Cell lines

The WTC11 human induced pluripotent stem cell line was obtained from Dr. Bruce Conklin’s laboratory (Gladstone Institute, UCSF). The WTC11 cells were derived from adult skin fibroblasts, and there is no associated identifier linking the cells to the original donor. The WTC11 cells are also available from Coriell #GM25256 (RRID: CVCL_Y803).
