# Supplementary material for: Targeted CRISPR activation is functional in engineered human pluripotent stem cells but undergoes silencing after differentiation into cardiomyocytes and endothelium
Source: Cell Mol Life Sci. 2024 Feb 19;81(1):95. doi: 10.1007/s00018-023-05101-2 (PMC10876724; doi:10.1007/s00018-023-05101-2)
Supplement: Supplementary file 1 — Supplementary material 1 (DOCX 16515 kb) [file 18_2023_5101_MOESM1_ESM.docx]

**SUPPLEMENTAL FIGURES**

**
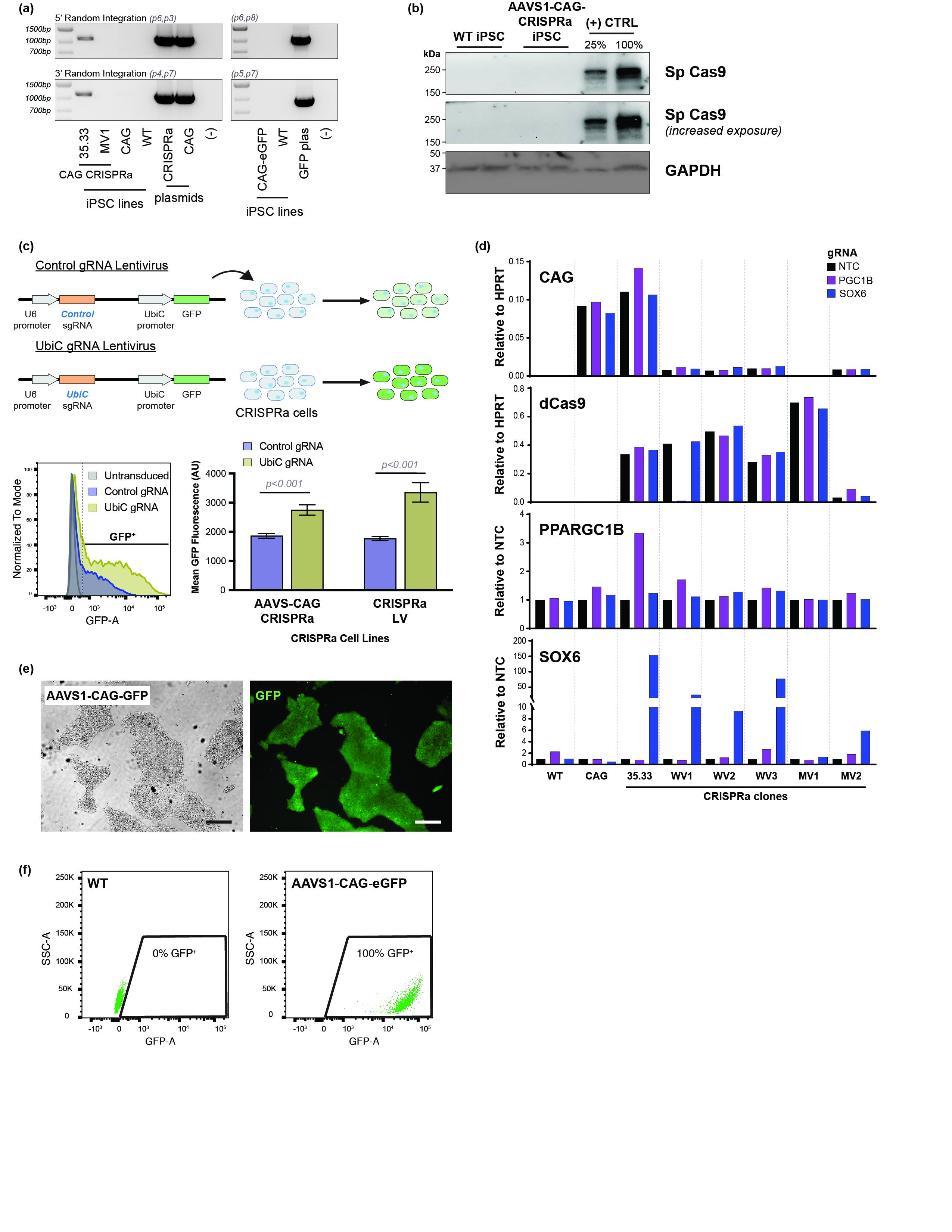
**

**Supplementary Fig. 1** Targeting of *AAVS1* locus. (a) Genotyping of *AAVS1*-targeted stem cell lines using primers to assess random integration of donor plasmids. (b) Western blot for dCas9-VPR in *AAVS1*-CAG-CRISPRa hiPSCs (Clone 35.33). Positive control lane is lysate from HEK293FT transfected with *AAVS1*-CAG-dCas9-VPR donor plasmid, 25% and 100% of total loaded protein. (c) To measure CRISPRa activity, cells are transduced with lentiviruses expressing GFP driven by a weak ubiquitin C (UbiC) promoter. These constructs also express a gRNA sequence, either control or specific for the UbiC promoter. If dCas9-VPR is present and active in cells, it will utilize the UbiC gRNA to upregulate GFP expression; GFP intensity is measured by flow cytometry to assess dCas9-VPR function. WTC11 CRISPRa hiPSCs, stably engineered at *AAVS1* locus (*AAVS1*-CAG-CRISPRa) or transduced with a CRISPRa lentivirus (CRIPSRa LV), were transduced with gRNA and activity was assayed by flow cytometry for GFP. Two biological replicates were performed and one representative replicate is shown. Error bars represent SEM. (d) Assessment of dCas9-VPR activity across multiple hiPSC CRISPRa clones. Different clones were transduced with control (NTC), PPARGC1B, or SOX6 gRNAs and harvested for gene expression analysis 4 days post-transduction. (e) Brightfield and fluorescent images of WTC11 *AAVS1*-CAG-eGFP hiPSCs. *Scale bar: 200μm.* (f) GFP expression measured by flow cytometry in *AAVS1*-CAG-eGFP stem cells.

**
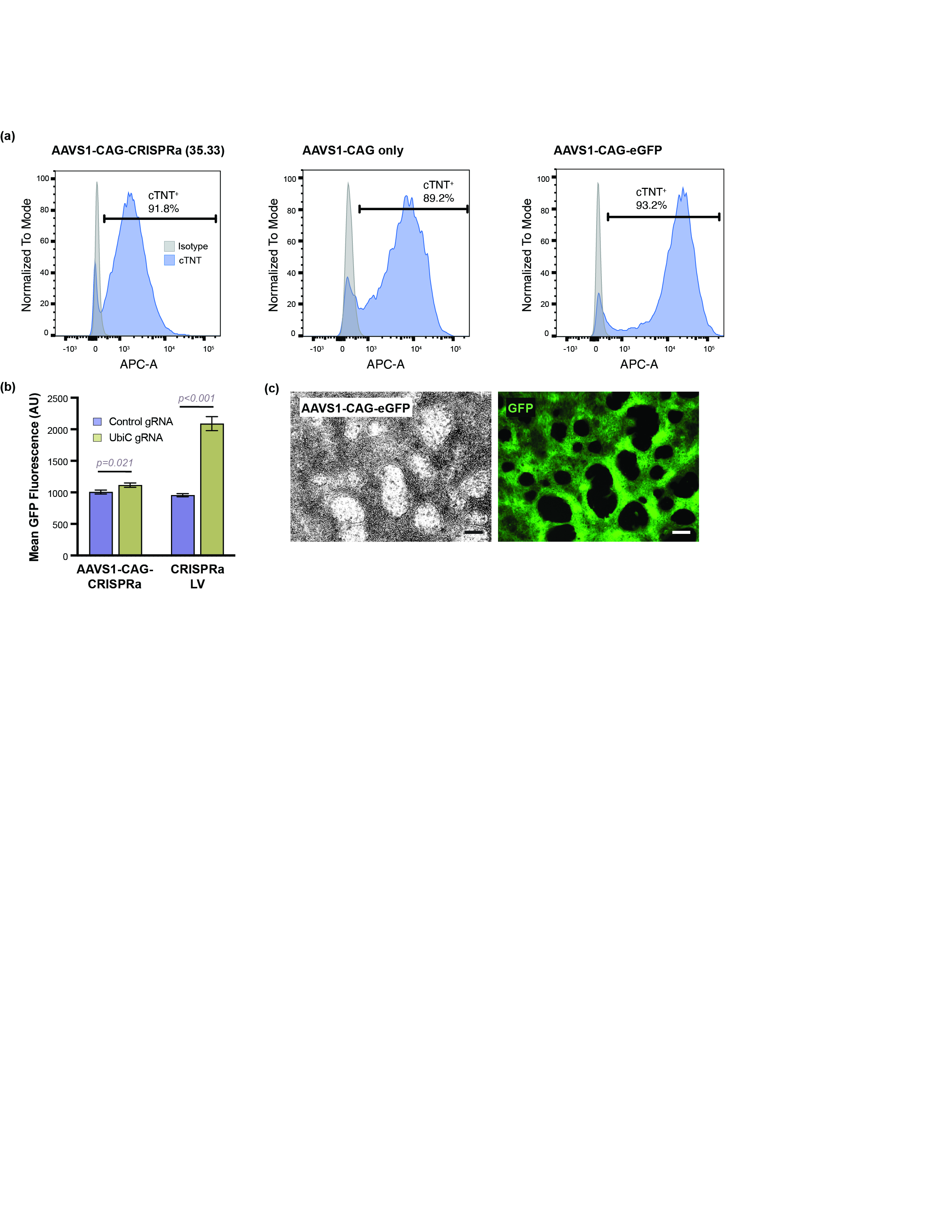
**

**Supplementary Fig. 2** *AAVS1*-targeted hiPSC-derived cardiomyocytes. (a) Flow cytometry analysis of cardiac troponin T (cTNT) cell population in Day 14 hiPSC-CMs. (b) CRISPRa activity measured using GFP reporter assay in hiPSC-CMs (*Refer to Suppl Fig 1c*). *AAVS1*-CAG-CRISPRa hiPSC-CMs (Day 14), containing integrated dCas9-VPR, were transduced with control or UbiC gRNA and UbiC-driving GFP reporter. Mean GFP fluorescence intensity was measured by flow cytometry to assess dCas9-VPR activity. Representative data, of two biological replicates, is shown. Error bars represent SEM. (c) Brightfield and fluorescent images of WTC11 *AAVS1*-CAG-eGFP hiPSC-CMs. *Scale bar: 200μm.*

**
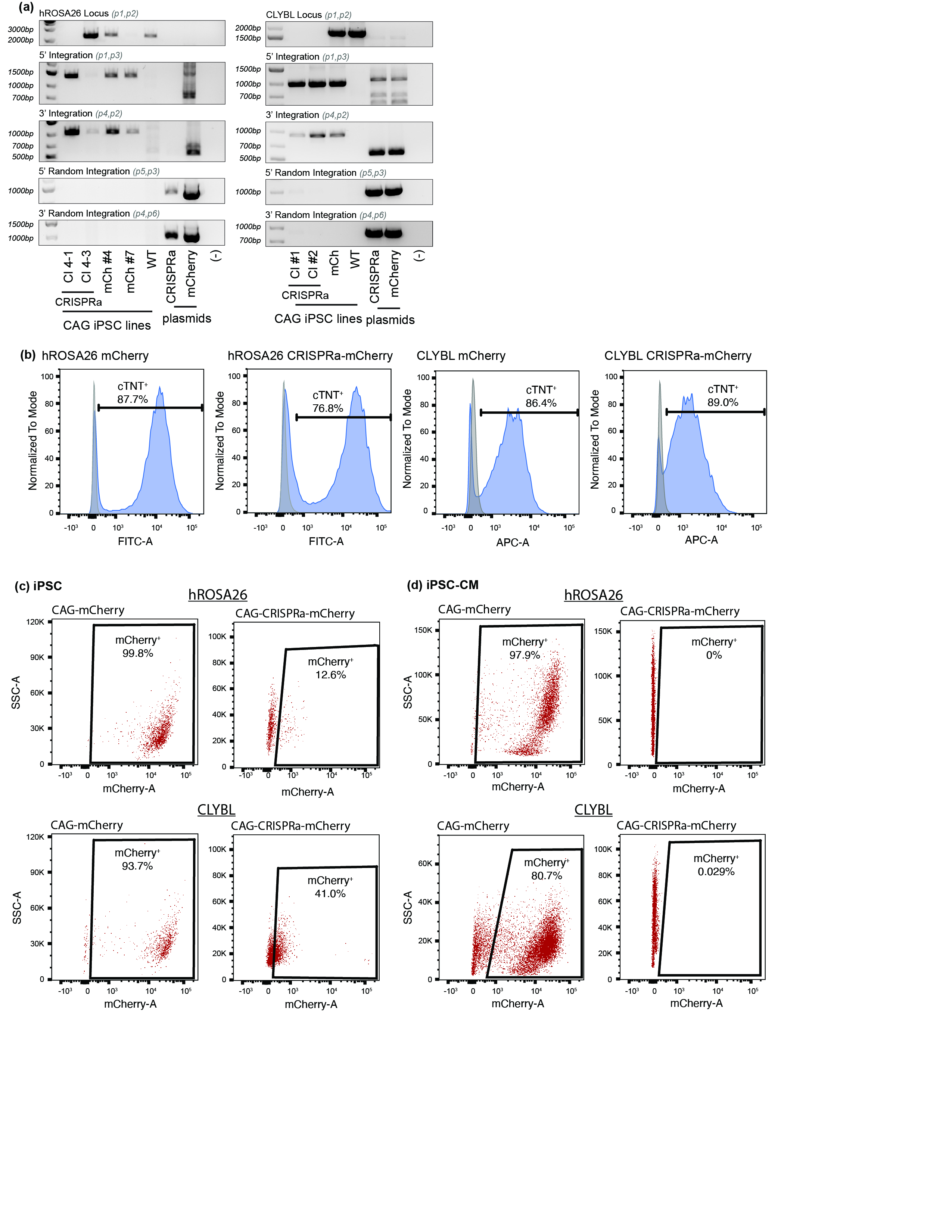
**

**Supplementary Fig. 3** *hROSA26* and *CLYBL* targeting. (a) Genotyping of CRISPRa-mCherry and mCherry cell lines with targeting at human *ROSA26* and *CLYBL* safe harbor sites. Primers used are indicated and target locations are shown in Fig 3a. Genomic DNA from wild type (WT) hiPSCs and donor plasmids used for cell line generation were used as control template DNA. (b) Cardiac troponin T flow cytometry analysis of *hROSA26*- and *CLYBL*-targeted Day 14 hiPSC-CMs. mCherry expression in (c) hiPSCs and (d) hiPSC-CMs. Gating was determined according to wild type unedited WTC11 cells.

**
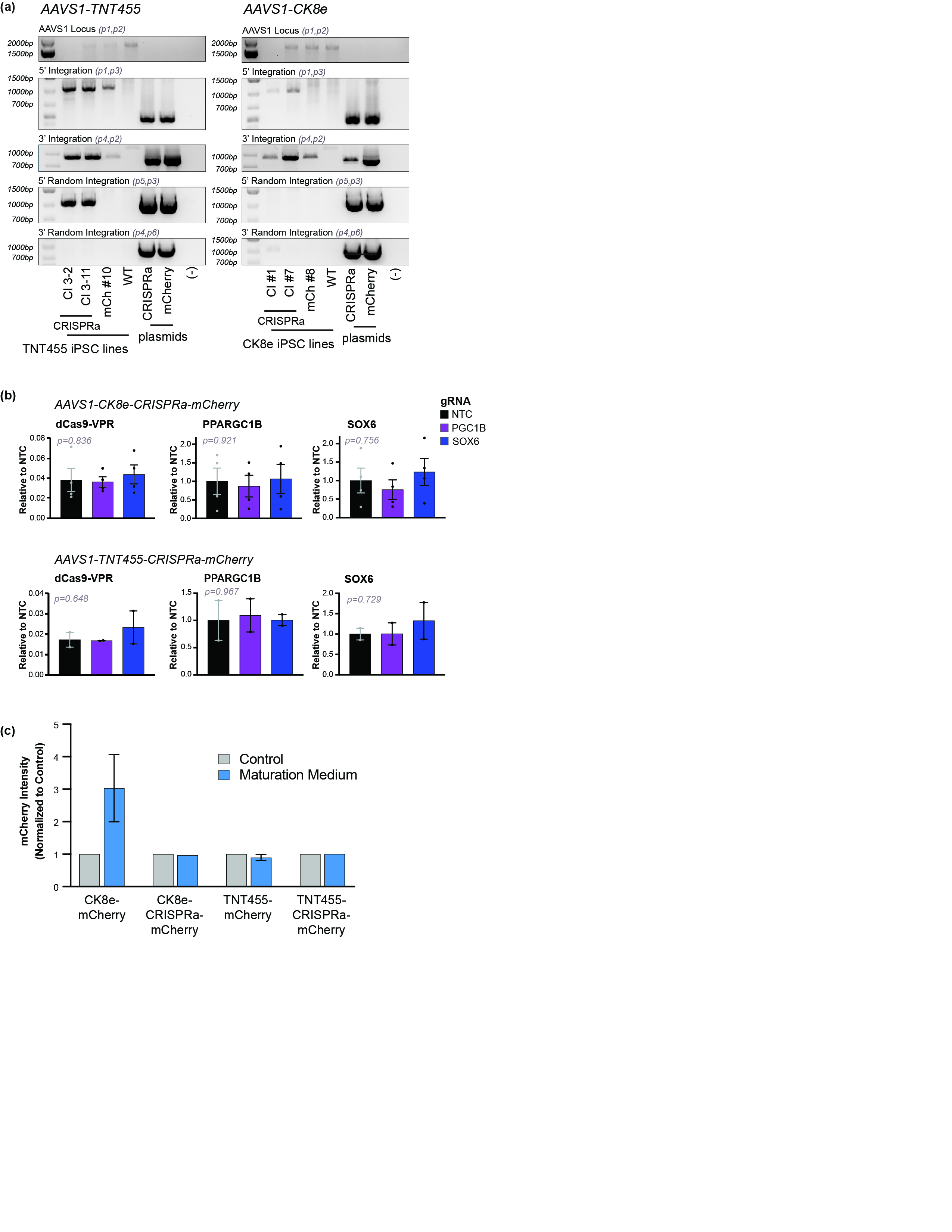
**

**Supplemental Fig. 4** Engineered cell lines with muscle-specific regulatory cassettes. (a) Genotyping of WTC11 cells engineered at *AAVS1* locus with muscle regulatory cassettes. Genomic DNA from wild type (WT) hiPSCs and donor plasmids used for cell line generation were used as control template DNA. (b) Day 14 hiPSC-CMs were transduced with gRNA targeting the indicated genes and harvested after 1 week. mRNA expression of target genes were measured by quantitative RT-PCR. dCas9-VPR expression is normalized to HPRT and target gene expression is normalized to non-targeting control samples. One-way ANOVA was performed to calculate statistical significance. *n = 2-4 independent differentiations.* (c) Day 15 hiPSC-CMs were cultured in maturation medium (low glucose supplemented with T3 and dexamethasone) to further mature cells for 7 days. mCherry expression was assessed by flow cytometry. mCherry geometric mean intensity of mCherry-positive cells is representative relative to respective controls. All error bars represent SEM.

**Supplemental Table 1. Genotyping Primers**

| ***AAVS1* Safe Harbor** | | |
| --- | --- | --- |
| ***CAG / CAG-CRISPRa*** | **Sequence** | **Expected Band (bp)** |
| AAVS1 Locus | CTGTTTCCCCTTCCCAGGCAGGTCC | 1693 |
|  | TGCAGGGGAACGGGGCTCAGTCTGA |  |
| 5’ Integration | CTGTTTCCCCTTCCCAGGCAGGTCC | 1032 |
|  | GTGCCCAGTCATAGCCGAAT |  |
| 3’ Integration | CCTCCCCCTGAACCTGAAAC | 2151 |
|  | TGCAGGGGAACGGGGCTCAGTCTGA |  |
| 5’ Random Integration | TAACGCCAGGGTTTTCCCAG | 1060 |
|  | GTGCCCAGTCATAGCCGAAT |  |
| 3’ Random Integration | CCTCCCCCTGAACCTGAAAC | 1077 |
|  | ATGCTTCCGGCTCGTATGTT |  |
|  | | |
| ***CAG -eGFP*** | **Sequence** | **Expected Band (bp)** |
| 5’ Integration | CTGTTTCCCCTTCCCAGGCAGGTCC | 1032 |
|  | GTGGGCTTGTACTCGGTCAT |  |
| 3’ Integration | GCGGATCGACAGTACTAAG | 2151 |
|  | TGCAGGGGAACGGGGCTCAGTCTGA |  |
| 5’ Random Integration | TAGTCCTGCAGGTTTAAACG | 1060 |
|  | GTGGGCTTGTACTCGGTCAT |  |
| 3’ Random Integration | GCGGATCGACAGTACTAAG | 1077 |
|  | AGGGCGAATTGAATTTAGCG |  |
|  | | |
| ***CK8e- & TNT455-mCherry / CRISPRa*** | **Sequence** | **Expected Band (bp)** |
| 5’ Integration | CTGTTTCCCCTTCCCAGGCAGGTCC | 1032 |
|  | GTGCCCAGTCATAGCCGAAT |  |
| 3’ Integration | CACTGCCTTGACAGTACTCT | 893 |
|  | TGCAGGGGAACGGGGCTCAGTCTGA |  |
| 5’ Random Integration | TAACGCCAGGGTTTTCCCAG | 1060 |
|  | GTGCCCAGTCATAGCCGAAT |  |
| 3’ Random Integration | CACTGCCTTGACAGTACTCT | 955 |
|  | ATGCTTCCGGCTCGTATGTT |  |

| ***hROSA26* Safe Harbor** | | |
| --- | --- | --- |
| ***CAG-mCherry / CAG-CRISPRa*** | **Sequence** | **Expected Band (bp)** |
| hROSA26 Locus | GAGAAGAGGCTGTGCTTCGG | 2186 |
|  | ACAGTACAAGCCAGTAATGGAG |  |
| 5’ Integration | GAGAAGAGGCTGTGCTTCGG | 1274 |
|  | AAGACCGCGAAGAGTTTGTCC |  |
| 3’ Integration | GAGAATAGCAGGCATGCTG | 1020 |
|  | ACAGTACAAGCCAGTAATGGAG |  |
| 5’ Random Integration | CGTTGTAAAACGACGGCCAG | 1148 |
|  | GTGCCCAGTCATAGCCGAAT |  |
| 3’ Random Integration | GAGAATAGCAGGCATGCTG | 931 |
|  | AGGAAACAGCTATGACCATG |  |

| ***CLYBL* Safe Harbor** | | |
| --- | --- | --- |
| ***CAG-mCherry / CAG-CRISPRa*** | **Sequence** | **Expected Band (bp)** |
| *CLYBL* Locus | TAAGTGACCCCTGGCGAGAC | 1728 |
|  | AGATGGAGCAGTGGATGACA |  |
| 5’ Integration | TAAGTGACCCCTGGCGAGAC | 962 |
|  | AAGACTTCCTCTGCCCTCTC |  |
| 3’ Integration | TCCAGGACGGAGTCAGTGAG | 907 |
|  | AGATGGAGCAGTGGATGACA |  |
| 5’ Random Integration | CACAGGAAACAGCTATGACC | 980 |
|  | AAGACTTCCTCTGCCCTCTC |  |
| 3’ Random Integration | TCCAGGACGGAGTCAGTGAG | 874 |
|  | TTTTCCCAGTCACGACGTTG |  |

**Supplemental Table 2. gRNA Sequences**

| **Target Sequence** | **gRNA gBlock for cloning (gRNA sequence highlighted)** |
| --- | --- |
| NTC_g1 | atcttgtggaaaggacgaaacacc**GACGGAGGCTAAGCGTCGCAA**gtttaagagctatgctggaaacagcatagcaagt |
| NTC_g2 | atcttgtggaaaggacgaaacacc**GCGCTTCCGCGGCCCGTTCAA**gtttaagagctatgctggaaacagcatagcaagt |
| PPARGC1B_Spy_g1 | atcttgtggaaaggacgaaacacc**GCCCCGCAGCTAGCGGCCCTG**gtttaagagctatgctggaaacagcatagcaagt |
| PPARGC1B_Spy_g2 | atcttgtggaaaggacgaaacacc**GCCGCAGGGCCGCTAGCTGCG**gtttaagagctatgctggaaacagcatagcaagt |
| PPARGC1B_Spy_g3 | atcttgtggaaaggacgaaacacc**GTGCCGCAGGGCCGCTAGCTG**gtttaagagctatgctggaaacagcatagcaagt |
| SOX6_Spy_g1 | atcttgtggaaaggacgaaacacc**GCTCCCCTCCCAGACAACAC**gtttaagagctatgctggaaacagcatagcaagt |
| SOX6_Spy_g2 | atcttgtggaaaggacgaaacacc**GCAAGATGAGACAAGAGGCG**gtttaagagctatgctggaaacagcatagcaagt |
| SOX6_Spy_g3 | atcttgtggaaaggacgaaacacc**GCACAGGCAAGATGAGACAAG**gtttaagagctatgctggaaacagcatagcaagt |

**Supplemental Table 3. qPCR Primers**

| **Gene** | **Sequence** |
| --- | --- |
| HPRT | TGACACTGGCAAAACAATGCA |
|  | GGTCCTTTTCACCAGCAAGCT |
| CAG | CTGACTGACCGCGTTACTCC |
|  | GCCAAAATGATGAGACAGCACA |
| dCas9-VPR | GTGTGCCAGCCAAAACGAAT |
|  | CACAGTATCGGCCATCTCCC |
| Neomycin | CGACCACCAAGCGAAACAT |
|  | CTCTTCGTCCAGATCATCCTGAT |
| PPARGC1B | GATGCCAGCGACTTTGACTC |
|  | ACCCACGTCATCTTCAGGGA |
| SOX6 | AGGGAGTCTTGCCGATGTG |
|  | CAGGCTCTCAGGTGTACCTTTA |

**Supplemental Table 4. Antibodies**

| **Flow Cytometry** | | |
| --- | --- | --- |
| **Target** | **Vendor** | **Catalog Number** |
| PerCP-Cy5.5 Mouse anti-OCT3/4 | BD Pharmingen | 560794 |
| PerCP-Cy5.5 Mouse IgG1 | BD Pharmingen | 552834 |
| Cardiac Troponin T APC | Miltenyi Biotec | 130-120-403 |
| REA Control Antibody IgG1 APC | Miltenyi Biotec | 130-113-446 |

| **Western** | | |
| --- | --- | --- |
| **Target** | **Vendor** | **Catalog Number** |
| Sp Cas9 | Diagenode | C15200203 (Ms) |
| GAPDH | Abcam | ab8245 (Ms) |
| PGC1B | Abcam | ab176328 (Rb) |
| Goat anti-Mouse IgG HRP | Life Technologies | 62-6520 |
| Goat anti-Rabbit HRP IgG | Life Technologies | 65-6120 |

**Supplemental Table 5. Bisulfite Methylation-Specific PCR Primers**

| **Region** | **Gene** | **Sequence** |
| --- | --- | --- |
| Hypermethylated Region | CAG MSP (Methylated) | CGGTAGTTAATTAGAGCGGC |
|  |  | GCTCACCTATAAAAATAACGCGA |
|  | CAG MSP (Unmethylated) | GGTGGTAGTTAATTAGAGTGGTG |
|  |  | CAATCAATCAAAACCAAAACAAAC |
| Differentially Methylated Region | CAG MSP (Methylated) | CGGGGTTTTGTGCGTTTC |
|  |  | AACCGAACCGTACTCAACAACTC |
| Intronic Region | CAG BSP | GGATTTTTTTTGTTTTAAATTTGTG |
|  |  | ATAATAAAACAACACAATAACCAACA |

| **HpaII Analysis** | **Sequence** |
| --- | --- |
| HpaII Site Primer Set 1 | CTTTGTGCGCTCCGCAGTG |
|  | CGCACGCAGCCTTTGTTCC |
| HpaII Site Primer Set 2 | CTGTGGCTGCGTGAAAGCC |
|  | CGCACGCAGCCTTTGTTCC |
| Control Region (mCherry cell line) | TACAGCTCCTGGGCAACGTG |
|  | TTGGTCACCTTCAGCTTGG |
| Control Region (CRISPRa cell line) | TACAGCTCCTGGGCAACGTG |
|  | CGGCACCTTGTACTCGTCCG |
